# Supplementary material for: Empirical methods for controlling false positives and estimating confidence in ChIP-Seq peaks
Source: BMC Bioinformatics. 2008 Dec 5;9:523. doi: 10.1186/1471-2105-9-523 (PMC2628906; doi:10.1186/1471-2105-9-523)
Supplement: Additional File 1 — A variety of html documents from the USeq web site detailing the available applications, their best usage, output file type descriptions, command line menus, etc. [file 1471-2105-9-523-S1.zip › USeqUserGuides/JavaDoc.zip!/JavaDoc/index-files/index-13.html]

N-Index


|  |  |  |
| --- | --- | --- |
| **Overview** \| Package \| Class \| Use \| **Tree** \| **Deprecated** \| **Index** \| **Help** | |  |
| **PREV LETTER**   **NEXT LETTER** | **FRAMES**    **NO FRAMES**     **All Classes** |  |


A B C D E F G H I J L M N O P Q R S T U V W X Z 

---


## **N**

**NAME\_NUM\_REAL\_BASES\_FILE** - Static variable in class util.bio.seq.ConvertFasta2RealSeqBoolean: **nameParser** - Static variable in class util.bio.seq.IndexedSequence: **nearestNeighborOligoTm(String, double, double)** - Static method in class util.bio.calc.NucleicAcid: Calculates nearest neighbor tm given a DNA oligonucleotide. **NEBParser** - Class in util.bio.digest: Parses a New England Biolabs restriction enzyme file into an array of `Enzyme`s. **NEBParser()** - Constructor for class util.bio.digest.NEBParser: **NewAntibody** - Class in bioroot.antibody: **NewAntibody()** - Constructor for class bioroot.antibody.NewAntibody: **NewOligo** - Class in bioroot.oligo: **NewOligo()** - Constructor for class bioroot.oligo.NewOligo: **NewPlasmid** - Class in bioroot.plasmid: **NewPlasmid()** - Constructor for class bioroot.plasmid.NewPlasmid: **NewStrain** - Class in bioroot.strain: **NewStrain()** - Constructor for class bioroot.strain.NewStrain: **newUseDilutionIndex(AntibodyBean, DBUtil, HashMap)** - Static method in class bioroot.antibody.AntibodyBase: Scans a hashmap for a newUseDilution selection, returns the index number **nonAmbiguousLetters** - Static variable in class util.gen.Passwords: **NormalizeAgilentData** - Class in expr: Takes two replicas, parses out those probes that are not 1's, median centers, then averages ProbeName ProbeName LogRatio gIsPosAndSignif rIsPosAndSignif LogRatio gIsPosAndSignif rIsPosAndSignif A\_51\_P100021 A\_51\_P100021 -0.087965 1 1 0.344989 0 1 A\_51\_P100034 A\_51\_P100034 0.038444 1 1 0.034538 1 1 A\_51\_P100052 A\_51\_P100052 -0.146955 1 0 0 0 0 **NormalizeAgilentData(String[])** - Constructor for class expr.NormalizeAgilentData: **normalizeCelFiles()** - Method in class trans.main.T2: **normalizeQuantileArrayScores(Quantile[][])** - Static method in class trans.cel.QuantileNormalization: Takes arrays of Quantile, and averages the values for a given array index. **normScrLookUpTable(int)** - Static method in class edu.utah.seq.analysis.ScanSeqs: Make a look up table for (t-c)/(Math.sqrt(t+c) returning tc[t][c]. **NucleicAcid** - Class in util.bio.calc: Nucleic Acid parameter calculator and converter static methods. **NucleicAcid()** - Constructor for class util.bio.calc.NucleicAcid: **nullArrays()** - Method in class util.gen.LinearRegression: Nulls the x and y arrays. **nullIndexedSequences()** - Method in class util.bio.seq.FetchIndexedSequence: Good to call after each call to fetchSequences() unless you'll be fetching more from the same chromosome. **nullIntensityArrays()** - Method in class trans.qc.CelFileStats: Nulls the virtualCel and collapsed intensities array. **nullPositionScoreArrays()** - Method in class edu.utah.seq.data.PointData: Nulls the position and score arrays to save memory. **Num** - Class in util.gen: Math and statistical static methods. **Num()** - Constructor for class util.gen.Num: **numberBases(BindingRegion[])** - Static method in class trans.anno.IntersectRegions: **numberBases(BindingRegion[])** - Static method in class util.bio.parsers.FetchGenomicSequences: **numberBinHitsToLeftAndIncludingValue(double)** - Method in class util.gen.Histogram: Returns number of bin hits in the bins to the right of the value's bin including the numMoreThanMax. **numberBinHitsToRightAndIncludingValue(double)** - Method in class util.gen.Histogram: Returns number of bin hits in the bins to the right of the value's bin including the numMoreThanMax. **numberBinHitsToRightOfValue(double)** - Method in class util.gen.Histogram: Returns number of bin hits in the bins to the right of the value's bin including the numMoreThanMax. **numberFilesExist(File, String)** - Static method in class util.gen.IO: Returns the number of files that end with the given extension. **numberFilesExist(File[])** - Static method in class util.gen.IO: Returns the number of files that exist in the array. **numberLetters** - Static variable in class util.gen.Passwords: **numberOfIntervalsToMake()** - Method in class trans.main.T2: Returns the number of intervals that remain to be made. **numberReplicasToMake()** - Method in class trans.main.T2: **numberUniqueValues(int[], int, int)** - Static method in class trans.tpmap.WindowMaker: Given a sorted set of values, a start and stop index (inclusive) returns the number of unique values.

---


|  |  |  |
| --- | --- | --- |
| **Overview** \| Package \| Class \| Use \| **Tree** \| **Deprecated** \| **Index** \| **Help** | |  |
| **PREV LETTER**   **NEXT LETTER** | **FRAMES**    **NO FRAMES**     **All Classes** |  |


A B C D E F G H I J L M N O P Q R S T U V W X Z 

---
